# Impact of High-Density Urban Built Environment on Chronic Obstructive Pulmonary Disease: A Case Study of Jing’an District, Shanghai

**DOI:** 10.3390/ijerph17010252

**Published:** 2019-12-30

**Authors:** Lan Wang, Rui Chen, Wenyao Sun, Xiaoming Yang, Xinhu Li

**Affiliations:** 1 College of Architecture and Urban Planning, Tongji University, 1239 Siping Road, Shanghai 200092, China; wanglan@tongji.edu.cn (L.W.); 1630043@tongji.edu.cn (W.S.); 2 Institute of Engineering and Industry, Tongji University, 1239 Siping Road, Shanghai 200092, China; chenrui_tongji@163.com; 3 Jing’an District Center for Disease Control and Prevention, Shanghai 200072, China

**Keywords:** built environment, COPD, respiratory health, geographically weighted regression

## Abstract

Respiratory health is a focus of interdisciplinary studies involving urban planning and public health. Studies have noted that urban built environments have impacts on respiratory health by influencing air quality and human behavior such as physical activity. The aim of this paper was to explore the impact of urban built environments on respiratory health, taking chronic obstructive pulmonary disease (COPD) as one of the typical respiratory diseases for study. A cross-sectional study was conducted including all cases (N = 1511) of death from COPD in the high-density Jing’an district of Shanghai from 2001 to 2010. Proxy variables were selected to measure modifiable features of urban built environments within this typical high-density district in Shanghai. A geographically weighted regression (GWR) model was used to explore the effects of the built environment on the mortality of COPD and the geographical variation in the effects. This study found that land use mix, building width-height ratio, frontal area density, and arterial road density were significantly correlated to the mortality of COPD in high-density urban area. By identifying built environment elements adjustable by urban planning and public policy, this study proposes corresponding environmental intervention for respiratory health.

## 1. Introduction

Chronic obstructive pulmonary disease (COPD) is a major chronic respiratory disease, which ranked the third among all causes of death in 2016 according the World Health Organization (WHO) [1]. The prevalence of COPD among population ages 20–39 and over 40 were 8.6% and 13.7%, respectively, in China [2]. COPD, with airway and/or alveolar abnormality, is characterized by persistent respiratory symptoms and airflow limitation [3]. COPD is also characterized by a slow and sustained development. There are many factors that threaten the death of patients throughout the whole course of the disease. 

Epidemiological studies have demonstrated that the development of COPD is mainly affected by personal characteristics (e.g., genetics, age, gender, and smoking status), exposure to air pollution, socioeconomic status, and physical activity [3,4,5,6,7]. Sudden cardiac death, heart failure, respiratory failure, chronic hypoxia and carbon dioxide retention, increased pulmonary artery pressure, and chronic impairment of lung function are risk factors of death for COPD patients [8,9,10,11,12], and the damage of COPD to human organs increases with aging [12,13,14,15]. Death from COPD presents its significant association with air pollutants [16,17]. The human exposure to air pollution is associated with increased mortality [18]. A high prevalence of COPD was reported in urban areas, especially in high-density areas [19,20,21]. The possible effects of urban built environments on COPD, therefore, may function through two ways: air quality and physical activity. First, urban built environments could influence the concentration and distribution of atmospheric particle matters by specific spatial factors. It includes: (a) land use (mixed land use [22], land utilization type [23]), (b) road traffic (vehicle emissions [24,25,26], dwelling distance [27], airflow at road intersection [28,29], isolated green belt along road [30]), (c) green space and open space (single building with complex outdoor space [31,32,33], green coverage rate and green space types [34], plant leaves with dust effect [35], landscape types and distribution of the surface composition [36]), (d) urban space morphology (block scale [37], valley street space form [22,29,37,38,39], urban ventilation form [40,41]). Second, physical activity can be regarded as an important mediator of the built environment affecting respiratory health. Daily physical activity can decrease the risk of developing COPD [42,43,44,45,46,47,48], as well as improve the life quality of COPD patients [49,50]. Available public service facilities, connected road networks, and walkable streets may promote physical activity of residents [51,52,53]. These urban built environmental exposures thus have impacts on the morbidity and modality of COPD. 

Most studies addressing the association between the environment and COPD have mainly focused on the variation of socio-economic environments [54] and natural environments such as altitude [55], temperature [56], and humidity [57]. Little has been reported yet on how urban-built environment exposure can influence individuals’ respiratory health. Understanding how modifiable features of urban built environments are associated with the morbidity and modality of respiratory diseases is important as the prevalence of COPD, asthma, and lung cancer are growing concerns to the public health.

The purpose of this study was to investigate the association between modifiable features of urban built environments and respiratory health using COPD as a case. We selected Jing’an District, one of the high-density districts in both population and buildings in Shanghai, as the study area. The geographically weighted regression (GWR) model was developed to identify risk factors of urban built environments on COPD and its geographical variation.

## 2. Materials and Methods

### 2.1. Study Area

**Figure 1:**
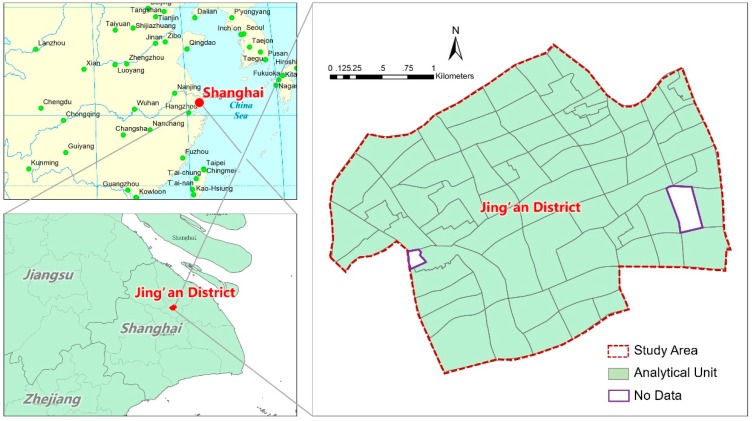
Location and space schematic diagram of the research scope.

Jing’an District, located in the central city of Shanghai, has a total population of 246,788 in 2010 (registered residence and people staying more than six months) and was 7.62 square kilometers before 2015. The population density is 32,387 capita per square kilometer in 2010 and 449 high-rise buildings over 11 floors in 2011 [58]. The jurisdiction of Residential Committee (*Juweihui*), usually called a neighborhood, was selected as spatial analytic unit. A total of 72 neighborhoods existed in Jing’an District before it was merged with another district in 2015. There were two outliers excluded from the analysis for their unavailable data (Figure 1).

### 2.2. Data

The COPD data were obtained from the vital registration database of the Center for Disease Control and Prevention (CDC) of Jing’an District. The database was coded according to the International Classification of Diseases, 10th Revision (ICD 10). Cases registered under J44 in the ICD 10 were regarded as COPD death cases. The information of all deaths from COPD in Jing’an District from 1 January 2001 to 31 December 2010 were extracted from the database, including age, gender, and residential address. 968 male cases and 543 female cases were included in this study. COPD death cases were matched to administrative units (Juweihui, neighborhood) based on residential addresses using Geocoding tools. In Jing’an District, the highest cumulative mortality of COPD was 10,148/100,000, while the lowest was 287/100,000. The average cumulative mortality of COPD was 2260/100,000.

The built environment data in this study were composed of three parts: (1) land use data were obtained from the Urban Planning and Land Resource Bureau of Jing’an District. These data presented the major use for each parcel of land as it was in the year of 2011. It was classified based on the national Code for Classification of Urban Land Use and Planning Standards of Development Land, including residential, administration and public services, commercial and business, etc.; (2) On-site data collection was conducted for each building for its height and style. We thus detailed the residential land according to its housing types, such as linong (2–3 floors), high-rise (11–24 floors), and workers’ new villages (4–6 floors). It also enabled the measurements to capture the wind situation within the neighborhood; (3) data for road systems were extracted based on land use data in 2011 through a process of vectorization. We also identified the level of each road including elevated highway, arterial, and branch roads. 

Existing studies have demonstrated the impact of a neighborhood’s demographic and economic status on respiratory health [59,60,61,62,63] and COPD [64,65,66]. We included the data about age structure, sex ratio, population education level, and occupation structure of each neighborhood based on the sixth national census in 2010.

### 2.3. Measures

**Figure 2:**
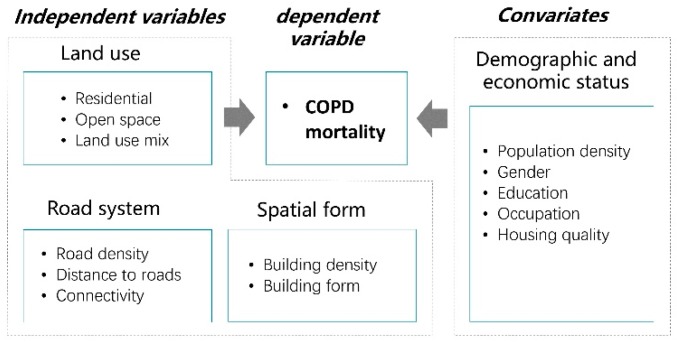
Conceptual framework.

**Figure A1:**
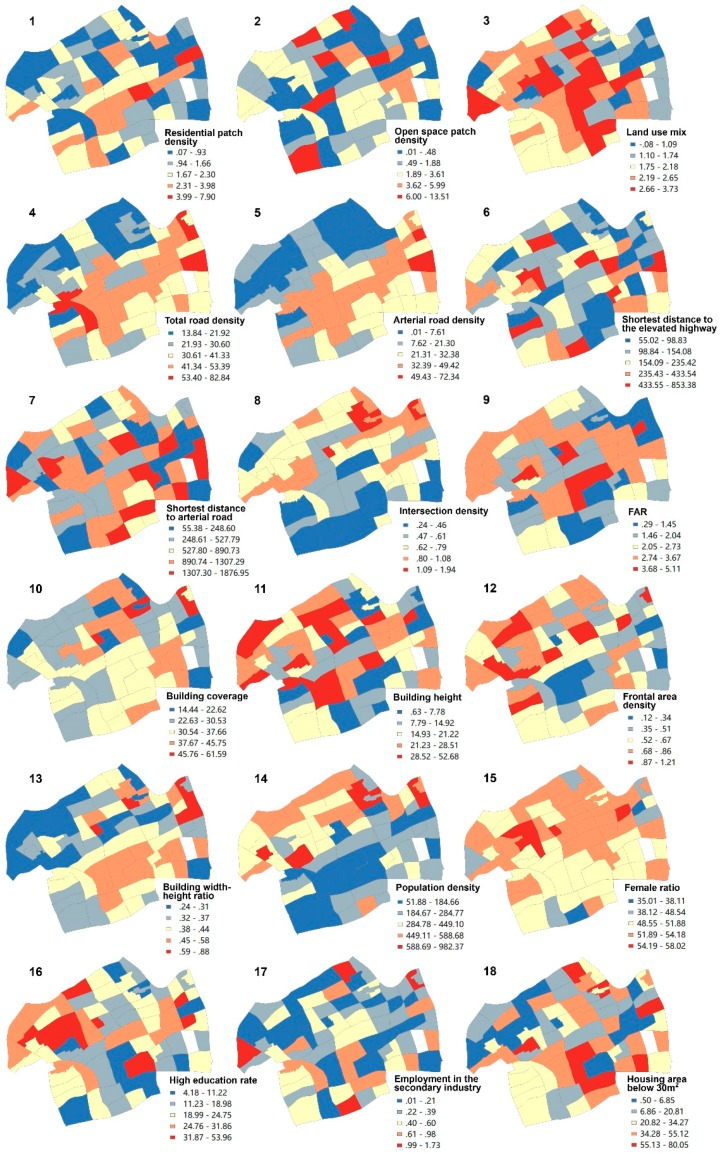
Spatial distribution of built environment elements.

To explore risk factors of urban built environments on COPD and its geographical variation, we employed the GWR model with the neighborhood COPD mortality as the dependent variable, three types of built environment factors as independent variables, and demographic and economic status as covariates (Figure 2). The neighborhood COPD mortality was calculated based on the data from the national census and the CDC. Built environment elements were selected according to their impact on air quality and physical activity, including land use, road system, and spatial form. The potential impact of all variables on respiratory health were listed in Table A1 and spatial distributions of built environment elements in Jing’an District were presented in Figure A1.

The cumulative mortality rate of COPD was employed because there are few death cases of COPD in neighborhoods per year. The formula is as follows:

mortality = Nc/Np × 100%,(1)

where Nc is the number of death cases from COPD in a neighborhood, and Np is the total population in a neighborhood. The unit of COPD mortality is the number of death cases per 100,000 population.

For land use, we selected patch density (PD) for residential and open space to measure its distribution. PD is an index to measure the fragmentation of land use. The larger the PD values, the greater it is fragmented. Current studies show that the land fragmentation increases residents’ exposure to air pollution. PD of residential and open space for each neighborhood was calculated using the following formula:

PD = N_L_/A, (2)

where N_L_ is the number of land parcels within the neighborhood, and A is the total area of this neighborhood. 

Land use mix has been identified as a significant indicator to promote physical activity [67], which captures the number of potential destinations within a certain area. It is calculated as:

(3) $$H=\overset{n}{\underset{i=1}{\sum }}\left(\frac{A_{i}}{A_{c}}\times \ln\frac{A_{i}}{A_{c}}\right)$$

where H is the level of land use mix of each neighborhood; n is the number of land use types, A_i_ is the land area of the ith type, and A_c_ is the total land area.

The second built environment element was road systems. Its measurement captured the level of air pollution generated from automobiles as one of the crucial risk factors for respiratory health. Dense road systems and short distances to heavy traffic roads increase the exposure to air pollutants. We employed road density and distance to the elevated highways and arterial roads as proxy variables. We adopted road systems instead of actual traffic volume to measure the level of air pollution generated from automobiles due to the limited data. Road systems could represent to a great extent the traffic level in our study because roads maintain consistent traffic volumes according to its capacity in this high-density urban district. Moreover, we used intersection density within a neighborhood, which may increase wind speed and thus decrease the concentration of air pollutants.

The third built environment element was spatial form. It attempted to capture the 3-Dimensional built environment variables and its impact, which were important and unique features for high density urban areas. The main criteria to select variables was the possible impact on wind speed and pollutants distribution. We used a total of five proxy variables to measure building density (floor area ratio, building coverage) and building form (building height, frontal area density, and building width-height ratio). Among them, floor area ratio (FAR) as a typical indicator to measure building mass was calculated by the total floor area of buildings within a parcel divided by its total land area. Building coverage was calculated by the first-floor area of buildings within a parcel divided by its total land area. 

**Figure A2:**
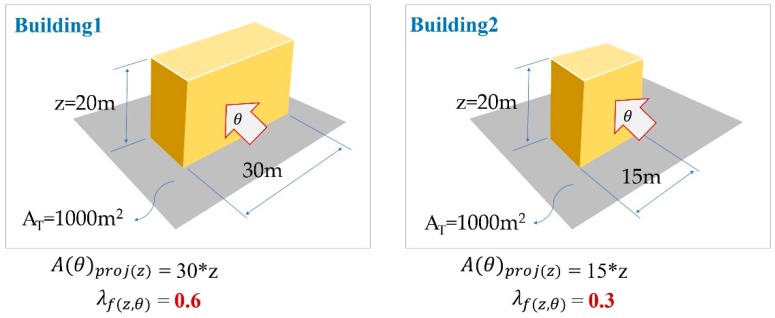
Measurement of frontal area density.

To capture the possible wind speed, the frontal area density was adopted as a proxy variable. It was calculated by the façade frontal area of a building to a certain wind direction divided by the total floor area of a neighborhood [68], as in the following formula:

(4) $$\lambda_{f\left(z,\theta \right)}=\frac{A{\left(\theta \right)}_{\mathrm{proj}\left(z\right)}}{A_{T}},$$

where A(θ)proj(z) is the façade frontal area of a building facing to a certain wind direction, θ is wind direction, AT is the total land area of a neighborhood, and z is the building height (see Figure A2).

The building width-height ratio also influences the wind speed and therefore the distribution of pollutants. It was calculated by the first-floor area of a building divided by its overall facade frontal area as in the following formula:

(5) $$\varepsilon =\sum_{i=1}^{n}\frac{A_{i}}{S_{i}*z},$$

where ε is the building width-height ratio, n is the number of buildings, Ai is the first-floor area of the ith building, Si is the perimeter of the ith building’s façade, z is the building height, and $S_{i}*z$ represents the overall frontal area of the ith building. 

Meanwhile, the demographic and economic status of a neighborhood were included as covariates because disparities may influence lifestyle, accessibility to health resources, and health outcomes. We included population density, gender, population with high education, occupation, and housing quality, which were measured by its corresponding proxy variables. Population density is the number of population divided by the area. Gender is the rate of females. Population with high education is the percentage of population with a bachelor’s degree above the total population. Occupation is the percentage of population engaged in manufacturing. Housing quality is the percentage of families with living areas less than 30 m^2^.

### 2.4. Modeling

We firstly used Spearman’s rank correlation to test the correlation between the mortality of COPD and the built environment variables. Then, we used a geographically weighted regression (GWR) model to unravel the impact of urban-built environments on the COPD mortality in high density areas. As an improvement on the traditional linear regression model and essentially a locally weighted least square method, the GWR model attempted to capture characteristics of specific spatial locations and its impact on the dependent variable. 

The geographically weighted regression model is shown as follows:

(6) $$y_{i}=\beta_{0}\left(u_{i},v_{i}\right)+\sum_{k=1}^{p}\beta_{k}\left(u_{i},v_{i}\right)x_{\mathrm{ik}}+\varepsilon_{i}\;(i=1,2,\ldots ,n),$$

where $\left(u_{i},v_{i}\right)$ is the longitude and latitude coordinates of the ith sampling point, and $\beta_{k}\left(u_{i},v_{i}\right)$ is the kth regression parameter at the ith sampling point.

We employed a stepwise regression process to identify significant variables. The explanatory variables were added to the GWR model step-by-step and the AICc of each model was compared so that the model with the lowest AICc was selected as the final model. Multicollinearity was measured by the variance inflation factor (VIF). No serious multicollinearity with a VIF > 10 was indicated in the GWR model. The GWR model with a Gaussian kernel form and a fixed, CV-optimized bandwidth was performed in ESRI ArcGIS 10.2 (ESRI, Redlands, CA, USA).

## 3. Results

### 3.1. Spearman’s Rank Correlation Analysis

The results of the Spearman’s rank correlation analysis were shown in Table 1. Land use mix was the only significant variable and showed negative correlation with COPD mortality in the land use category. As for road systems, both total road density and arterial road density were found to be positively correlated with COPD mortality. For spatial form, the frontal area density was negatively correlated with COPD mortality, while the building width-height ratio was positively correlated with COPD mortality. The population density had a negative correlation with COPD mortality, while other demographic and economic variables had no significant correlation with COPD mortality.

### 3.2. GWR

**Figure 3:**
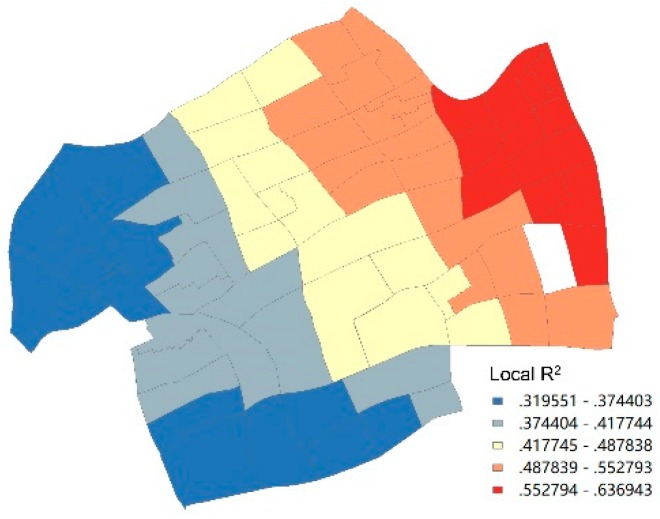
Local R^2^ Value distribution.

The overall R^2^ and adjusted R^2^ of the model, which reflected the fitness of the geographical weighted regression, was 0.644 and 0.552, respectively. The local R^2^ ranged from 0.32 to 0.64 from the west to the east of Jing’an District. This indicated that the local fitting degree of the model in the eastern neighborhoods was better than that in the western neighborhoods (Figure 3). 

**Figure 4:**
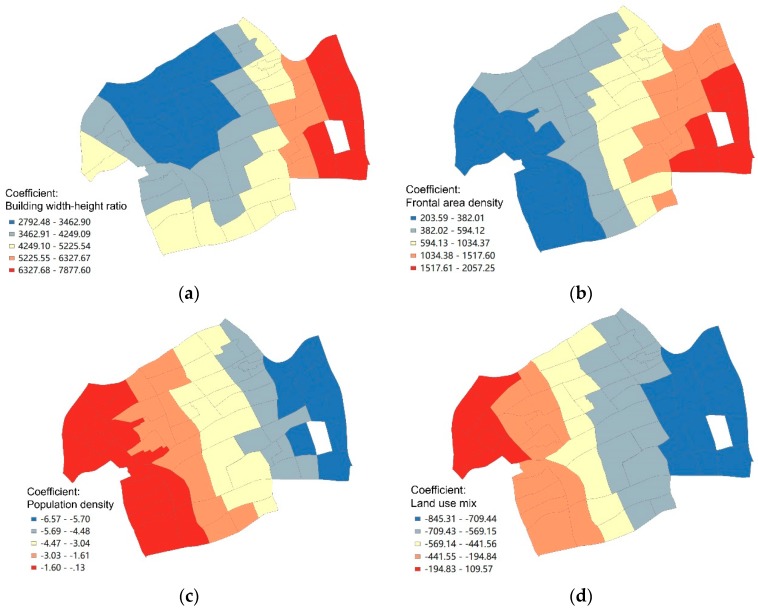

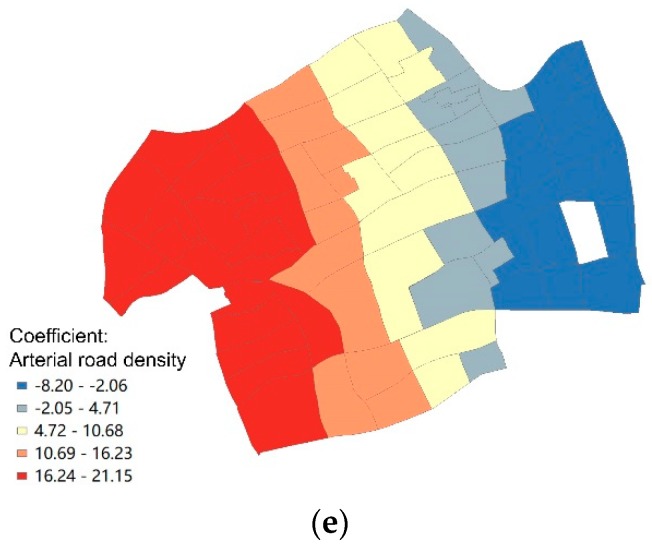
Spatial distribution of local coefficients of various significant influencing factors.

The results of the geographic weighted regression model illustrated that building width-height ratio was positively correlated with the mortality of COPD with the unstandardized coefficient changing with geographical location from 2792.48 to 7877.60 (Figure 4a). Moreover, the neighborhoods with high values of the local coefficient of building width-height ratio were located in the east of the district, which was matched to the spatial units with good model fittings. Frontal area density was also positively correlated to the mortality of COPD with the highest coefficient in the east of the district (Figure 4b). The unstandardized coefficient of frontal area density varied from 203.59 to 2057.25. In contrast, population density was negatively correlated to the mortality of COPD with the unstandardized coefficient from −6.57 to −0.13 (Figure 4c). The spatial distribution of the population density coefficient was basically consistent to that of local R^2^, in which high-value neighborhoods were in the east while low-value ones were in the west. The building width-height ratio, frontal area density, and population density had a single direction of influence, though the magnitude of the coefficients varied geographically. 

The mortality of COPD was more sensitive to the changes in building width-height ratio, frontal area density, and population density in the east district than in the west district. However, the influencing direction of land use mix and arterial road density varied geographically. The coefficient of land use mix ranged from −845.31 to 109.57, indicating that the association between land use mix and the mortality of COPD varied from a negative correlation to positive correlation (Figure 4d). Specifically, the land use mix was negatively correlated to the mortality of COPD in most neighborhoods except the northwest areas, in which the land use mix was positively correlated to the mortality of COPD. Arterial road density was also significantly correlated to the mortality of COPD with different directions. The coefficient of arterial road density ranged from −8.20 to 21.15 from east to west (Figure 4e). This indicated that the association between arterial road density and the mortality of COPD was negative in east neighborhoods and positive in west neighborhoods.

## 4. Discussion

Studies have noted that urban built environments have impacts on respiratory health by influencing air quality and human behavior such as physical activity [3]. The complexity of its impact, however, remains unraveled. We used COPD, one of the typical respiratory diseases, to explore its association to urban built environments. Geographical variation was also analyzed by developing a geographically weighted regression model, because built environmental elements may present different functions at different locations. With the strength of the GWR model, we identified significant built environmental elements and revealed geographical variation in terms of its magnitude and direction. Moreover, we selected proxy variables to measure modifiable features of urban built environments within a typical high-density district in Shanghai. By identifying built environmental elements adjustable by urban planning or public policy, we could improve respiratory health correspondingly. 

As an important land use indicator, most prior findings proved a positive effect of land use mix, meaning that a higher level of mixture would promote respiratory health [22]. This was in line with this study when we found that the mortality of COPD was negatively correlated to the land use mix in most neighborhoods. Land use mix providing multiple destinations within walkable distance would reduce the usage of motor vehicles and then decrease carbon emission and air pollution [69,70]. Physical activities such as walking and cycling would be also promoted. 

For road systems, we found arterial road density had a significant impact and geographical variation on the mortality of COPD. The magnitude of arterial road density’s influence on COPD decreased from west to east. Moreover, the correlation between arterial road density and the COPD mortality was positive in neighborhoods in the west, while it was negative in the eastern area. Two possible effects of arterial road density may be verified, including generating air pollutants and providing accessibility to healthcare facilities. Prior findings supported that myocardial infarction, heart and respiratory failure and their acute attacks could increase the death risk among COPD patients [9,10,12]. Emergency aid mainly depends on the accessibility to hospitals in China. At present, Chinese cities were developing 15-min first-aid circles in order to shorten the first-aid time and reduce mortality. For these reasons, the efficiency of ambulances has a great relationship with the traffic time and distance from the patient’s residence to the hospital. Due to the consistent heavy traffic on the road, the distance of medical facilities is particularly important in the high-density central city. In the western area with more hospitals, the effect of accessing healthcare facilities played a more significant role than that of generating air pollutants. On the contrary, air pollution caused by traffic on arterial roads outweighed the effect of healthcare facilities accessibility in the eastern neighborhoods. This finding contributed to the existing literature by revealing multiple effects of arterial roads, which could affect respiratory health in different ways in specific areas. The allocation of healthcare facilities such as hospitals need to consider road systems to support its emergency treatment. 

Among the variables of spatial form, we identified building form with proxy variables of frontal area density and building width-height ratio as significant variables instead of building density. Although the magnitude of correlation varied geographically, the direction of the effects was positive. Both of these two proxy variables measured the effect of building layout on the wind situation. The frontal area density was defined as the ratio of floor area to windward area of buildings. Low frontal area density was conducive for ventilation. It could accelerate the diffusion of particulate matter and then reduce its concentration [40,41]. Building width-height ratio measured the shape of buildings and its impact on wind speed. The higher the building width-height ratio, the slower the wind speed and the higher the concentration of air pollutants. It was also notable that the high-degree of correlation was located in the east of the district, which was also the low-density area for both building width-height ratio and frontal area density. This indicates that the mortality of COPD is likely to be more sensitive to the building form in low density areas. 

For demographic and economic status, we found that population density was negatively correlated to the mortality of COPD, which was inconsistent to most prior studies [61]. Previous studies that found a positive correlation between population density and mortality were often concluded by comparing the differences between urban and rural areas. It may be different because we examined the population density in the extremely high urbanized areas. The potential explanation for this result may be that the area of high population density in Jing’an District was associated with high-rise residential building developed after 1990s with better housing quality. The area of low population density was occupied with linong and workers’ new village with relevant poor housing quality and infrastructure. 

The findings of the GWR model identified not only the significant variables but also its geographical variation. It provided opportunity to explore multiple effects of built environment within a specific context. The association between the respiratory health and land use mix, arterial road density, building form and demographic features identified in this study led to the rethinking of principles of urban planning and policy making. 

The first limitation of this study is the lack of key information about smoking and genetics, which may affect the analysis results. The second limitation is that we are unable to capture the dynamic changes of the environment with the times, so that the variation in the temporal effects of the built environment on respiratory health is ignored in this study. The third limitation is that the built environment mainly refers to living places in this study and the built environment of the residents’ workplace is not well considered. The impact of the built environment on respiratory health may be overestimated. 

## 5. Conclusions

This study contributes to a better understanding of the complex relationship between urban built environments and respiratory health. Land use mix, arterial road density, building form, and demographic features have geographically varied effects on COPD.

The findings can provide an empirical basis for environmental intervention in high-density areas. The mixed pattern of land use can be encouraged to promote physical activities such as walking and cycling and improve residents’ respiratory health. Both positive and negative effects of road network density should be considered in urban planning. The allocation of healthcare facilities such as hospitals need to consider road systems to support emergency treatment. For building form, we shall reduce its frontal area to increase wind speed for better distribution of air pollutants. With the same floor area, the narrow façade might be preferred for respiratory health. Building form affects the circulation of wind, especially in high-density areas. Special attention should be paid to the impact of new construction and urban development on the wind environment. 

Future studies shall include data about other risk factors such as smoking, genetics, and work exposure. The changes in built environment and real-time traffic volume to capture air pollution generated from automobiles should also be considered. Moreover, this study implied that the proximity to hospitals might be a possible reductive factor for COPD mortality. It is necessary to consider the accessibility to hospitals and parks within and outside the study area. Further research remains necessary on the accurate estimation of emergency time in different built environments. 

## Figures and Tables

**Table 1:** Spearman’s rank correlation test on the relationship between independent variables and COPD mortality.

Categories	Sub-Categories	Variables	Correlation Coefficient	Sig. (Double Side)
Land use	Residential	Residential patch density	−0.118	0.331
	Open space	Open space patch density	−0.055	0.648
	Land use mix	Land use mix	−0.247 *	0.040
Road systems	Road density	Total road density	0.482 **	0.000
		Arterial road density	0.535 **	0.000
	Distance to roads	Shortest distance to the elevated highway	0.025	0.838
		Shortest distance to arterial road	0.056	0.643
	Connectivity	Intersection density	−0.126	0.298
Spatial form	Building density	FAR (Floor area ratio)	−0.111	0.361
		Building coverage	−0.073	0.547
	Building form	Building height	−0.134	0.267
		Frontal area density	−0.460 **	0.000
		Building width-height ratio	0.408 **	0.000
Demographic and economic status	Population density	Population density	−0.540 **	0.000
	Gender	Female ratio	−0.121	0.317
	Education	High education rate	−0.143	0.237
	Occupation	Employment in the secondary industry	0.122	0.315
	Housing quality	Housing area below 30 m^2^	0.034	0.777

Note: * *p* < 0.05, ** *p* < 0.01.

## Appendix A

**Table A1:** Measurement and impact of the built environment: demographic and economic factors.

Categories	Sub-Categories	Variables	Unit	Definition	Potential Impact in Hypotheses
Land use	Residential	Residential patch density	piece/hm^2^	Number of residential land/neighborhood total land area	Higher density increases human exposure to air pollutants
	Open space	Open space patch density	piece/hm^2^	Number of open space land/neighborhood total land area	Higher density increases human exposure to air pollutants
	Land use mix	Land use mix	/	$H=\overset{n}{\underset{i=1}{\sum }}\left(\frac{A_{i}}{A_{c}}*ln\frac{A_{i}}{A_{c}}\right)$	Decrease the usage of automobile and thus reduce air pollution
Road system	Road density	Total road density	km/km^2^	Road length/neighborhood total land area	Increase traffic volume and its air pollution
		Arterial road density	km/km^2^	Arterial road length/neighborhood total land area ×1000	Increase traffic volume and its air pollution
	Distance to roads	Shortest distance to the elevated highway	m	Shortest distance to the elevated highway	The shorter distance implies more human exposure to air pollution
		Shortest distance to arterial road	m	Shortest distance to arterial road	The shorter distance implies more human exposure to air pollution
	Connectivity	Intersection density	quantity/hm^2^	Intersections number/neighborhood total land area	Increase wind speed and thus decrease the concentration of air pollutants.
Spatial form	Building density	FAR (Floor area ratio)	/	Above-ground building Area/parcel land area	High density increase the concentration of air pollutants
		Building coverage	%	Building area/parcel land area	High density increase the concentration of air pollutants
	Building form	Building height	/	Standard deviation of building height	High density increase the concentration of air pollutants
		Frontal area density	/	∑ (Frontal area of a building facing to a certain wind direction)/parcel land area	High density decrease wind speed and thus increase the concentration of air pollutants
		Building width-height ratio	/	Building area/frontal Area	High density decrease wind speed and thus increase the concentration of air pollutants
Demographic and economic status	Population density	Population density	persons/hm^2^	Total population/neighborhood total land area	Affect the morbidity and mortality
	Gender	Female ratio	%	Women/total population	Affect the morbidity and mortality
	Education	High education rate	%	Bachelor degree or above/total	Life style and knowledge affect the morbidity and mortality
	Occupation	Employment in the secondary industry	%	Production, transportation equipment operators/total employment	Work exposure increases the mortality and mortality
	Housing quality	Housing area below 30 m^2^	%	Average per unit living area less than 30 m^2^	Low housing quality increases the mortality and mortality
